# Exploring the Influence of a Pomegranate Extract on the Functionality of Healthy and Diseased Human Gut Microbiota: An In Vitro Study

**DOI:** 10.3390/molecules30071634

**Published:** 2025-04-06

**Authors:** Daniele Giuseppe Buccato, Adriana Delgado-Osorio, Lorenza Francesca De Lellis, Maria Vittoria Morone, Hammad Ullah, Luana Izzo, Sonia Lombardi, Alessandro Di Minno, Costanza Valentina Riccioni, Dafni Moriki, José Ángel Rufián-Henares, Maria Daglia

**Affiliations:** 1 Department of Pharmacy, University of Napoli Federico II, Via D. Montesano 49, 80131 Naples, Italy; danielegiuseppe.buccato@unina.it (D.G.B.); lorenzafrancesca.delellis@unina.it (L.F.D.L.); luana.izzo@unina.it (L.I.); sonia.lombardi@unina.it (S.L.); alessandro.diminno@unina.it (A.D.M.); 2 Department of Nutrition and Food Sciences, Institute of Nutrition and Food Technology, Biomedical Re-Search Centre, Universidad de Granada, 18071 Granada, Spain; adrianadelgado@ugr.es (A.D.-O.); jarufian@ugr.es (J.Á.R.-H.); 3 Department of Experimental Medicine, Section of Microbiology and Clinical Microbiology, University of Campania “L. Vanvitelli”, 80138 Naples, Italy; mariavittoria.morone@unicampania.it; 4 School of Pharmacy, University of Management and Technology, Lahore 54000, Pakistan; hammadrph@gmail.com; 5 CEINGE-Biotecnologie Avanzate, Via Gaetano Salvatore 486, 80145 Naples, Italy; 6 R&D Department, Esserre Pharma Srl, 00191 Rome, Italy; c.riccioni@esserrepharma.it; 7 Allergology and Pulmonology Unit, 3rd Pediatric Department, National and Kapodistrian University of Athens, 12462 Athens, Greece; dafnimoriki@yahoo.gr; 8 Instituto de Investigación Biosanitaria (ibs.GRANADA), Universidad de Granada, 18140 Granada, Spain; 9 International Research Center for Food Nutrition and Safety, Jiangsu University, Zhenjiang 212013, China

**Keywords:** pomegranate extract, polyphenols, in vitro digestion, in vitro fermentation, antioxidant capacity, short-chain fatty acids, urolithins

## Abstract

Pomegranate is recognized for its health benefits, primarily due to its polyphenols and metabolites, such as urolithins (Uro-A), produced via colonic fermentation of ellagic acid (EA). These compounds make pomegranate a functional food with the potential to modulate chronic disease risk factors and enhance gut health by modulating microbiota. The aims of this study were (1) to evaluate the effect of in vitro digestion and fermentation mimicking human digestive processes on the total phenolic content (TPC) and the antioxidant capacity of a standardized pomegranate extract (PE), (2) to assess the effect of the digested PE on the functionality of gut microbiota isolated from healthy and diseased subject fecal materials via short-chain fatty acid (SCFA) determination, and (3) to measure Uro-A production using UHPLC Q-Orbitrap HRMS. The in vitro digestion and fermentation processes resulted in a significant increase in the TPC, while the antioxidant capacity was considerably reduced. Following the in vitro digestion process, the TPC increased from 232 ± 16 to 1656 ± 34 g GAE/g of PE. Moreover, the TPC in the fermented samples was calculated as 6139 ± 458 g GAE/g for the microbiota of healthy adults and 8375 ± 1388 g GAE/g for the microbiota of healthy children, compared to 1657 ± 34 g GAE/g for the non-fermented samples. The PE exerted a modulatory effect on gut microbiota functionality, as reflected by an increasing concentration of SCFAs, especially lactic acid. Overall, these data suggest that pomegranate might contribute to gut health and could be a candidate for further studies in view of its possible use as a prebiotic ingredient. Further research, including clinical studies, is needed to confirm these findings and explore the potential application of pomegranate extract as a functional ingredient in nutraceuticals and functional foods aimed at improving gut health.

## 1. Introduction

Pomegranate (*Punica granatum* L., Punicaceae) is a fruit consumed worldwide, native to China, the Indian subcontinent, Afghanistan, and Iran, where historic texts claim that it has been grown in these regions for thousands of years. Nowadays, pomegranate cultivation is extended from Iran, over the Mediterranean region, to the Turkish borders of Europe, the American Southwest, California, and Mexico [1,2]. Data from the U.S. Department of Agriculture [3] indicate that pomegranate fruit (per 100 g) contains dietary fiber (4 g), essential minerals, including calcium (10 mg), iron (0.3 mg), magnesium (12 mg), phosphorus (36 mg), potassium (236 mg), sodium (3 mg), zinc (0.35 mg), copper (0.158 mg), manganese (0.119 mg), and selenium (0.5 µg), as well as a range of vitamins, such as ascorbic acid (10.2 mg), thiamine (0.067 mg), riboflavin (0.053 mg), niacin (0.293 mg), pantothenic acid (0.377 mg), pyridoxine (0.075 mg), folate (38 µg), choline (7.5 mg), alpha-tocopherol (0.6 mg), and phylloquinone (16.4 µg). Additionally, it provides monounsaturated fatty acids (0.093 g) and polyunsaturated fatty acids (0.079 g). Moreover, pomegranate fruits are rich in polyphenols, with hydrolysable tannins (i.e., gallotannins and ellagitannins) being the most abundant ones, followed by hydroxycinnamic acids, flavanols (epigallocatechin), flavanones (naringenin and hesperidin), flavonols (myricetin, quercetin, and kaempferol), flavones (apigenin and luteolin), anthocyanins (delphinidin, cyanidine, and pelargonidine), and dihydrochalcones (phloridzin) [4,5]. These pomegranate phytochemicals, which account for 92% of the antioxidant capacity associated with the fruit [6], may influence cell signaling pathways, scavenge free radicals, chelate metals, and modify gene expression, among other properties [7,8,9]. In biological systems, pomegranate polyphenols can disrupt free radical chain reactions by donating H^+^ to lipid peroxides [10]. Moreover, some compounds (i.e., punicalagin) have the ability to scavenge hydrogen peroxide (H_2_O_2_) by donating an electron from the phenolic group to H_2_O_2_ and converting it into H_2_O [11]. The ability of pomegranate extracts to reduce Fe^3+^ to Fe^2+^ further demonstrates their electron donor capacity, which is mainly dependent on the quantity of phenolic hydroxyl groups in each molecule [12]. A study showed that quercetin and punicalagin in pomegranate juice chelate intracellular iron, hence limiting the production of free radicals [11]. Pomegranate juice demonstrated the highest antioxidant potency as compared to other polyphenol-rich beverages such as orange juice, red wine, green and black tea, cranberry juice, black cherry juice, blueberry juice, and açaí juice [12]. Other interesting biological effects of pomegranate fruit include anti-inflammatory properties against diseases induced by chronic low-grade systemic inflammation (i.e., rheumatoid arthritis, asthma, chronic obstructive pulmonary disease, or inflammatory bowel disease), cardiometabolic disorders (obesity, diabetes mellitus, hypertension, hypercholesterolemia, and atherosclerosis), different types of cancer, microbial infections, and wound healing [6,13].

Ellagitannins, especially punicalagins, obtained from pomegranate byproducts suppress the growth of certain pathogenic bacteria, including *Pseudomonas aeruginosa*, *Clostridium* species, and *Staphylococcus aureus*. However, they can also promote the growth of commensal bacteria and the de novo synthesis of short-chain fatty acids (SCFAs) such as acetate, propionate, and butyrate [14,15]. Following absorption, SCFAs may be transported to different organs and alter numerous pathways associated with health and disease. Acetate and butyrate are mostly involved in lipid biosynthesis, whereas propionate is primarily involved in gluconeogenesis. In addition to their direct role as significant energy sources, SCFAs can regulate multiple biological host responses, mainly oxidative and inflammatory stresses [16,17]. Animal studies indicated activation of peroxisome proliferator-activated receptors (PPARs) by SCFAs, which control gene expression related to differentiation, metabolism, and cancer [18,19,20]. By activating PPARα, SCFAs may prevent the expression of proinflammatory mediators, including activator protein (AP)-1, signal transducer and activator of transcription (STAT), and nuclear factor kappa B (NF-κB). Inhibition of these molecules may downregulate the genetic transcription of tumor necrosis factor (TNF-α), interleukins (IL-1 and IL-6), and cyclooxygenase (COX) [21].

This study aimed to evaluate the in vitro impact of a whole fruit pomegranate extract, whose metabolic profile was studied in our previous research [4], on the functionality of gut microbiota obtained from the fecal material of different donors (adults and children, both healthy and diseased, including obese and celiac subjects) using simulated in vitro digestion and fermentation processes, designed to mimic digestion in the human oral, gastric, and intestinal tract.

## 2. Results

The TPC and antioxidant properties of pomegranate extracts were assessed before and after digestion and fermentation. The antioxidant activity was evaluated using ABTS+ and DPPH assays, which measure radical scavenging capacity, along with the FRAP assay, which determines the reducing potential. The combined use of these assays allowed for a more comprehensive characterization of the antioxidant potential of the pomegranate extract [22]. To identify statistically significant variations between different treatment conditions, the TPC, radical scavenging activity (DPPH and ABTS+), and reducing power (FRAP) were compared across samples subjected to digestion and fermentation with gut microbiota isolated from the fecal material of both healthy and diseased (celiac and obese) individuals, including both adults and children. Additionally, the influence of digested and fermented pomegranate extract on gut microbiota functionality was assessed by quantifying SCFAs using UHPLC-RID analysis. Lastly, the potential of gut microbiota from different donors to facilitate the biotransformation of pomegranate polyphenols into urolithins, particularly Uro-A, was analyzed through UHPLC Q-Orbitrap HRMS.

### 2.1. TPC Evaluation of Pomegranate Extract Before and After Digestion and Fermentation Processes

The Folin–Ciocalteu assay revealed a significant increase in polyphenol release from the pomegranate extract following in vitro simulated digestion compared to the non-digested samples (Table 1). Specifically, the total phenolic content (TPC) rose from 232 ± 16 to 1656 ± 34 g GAE/g after digestion (*p* < 0.0001). Further fermentation of these digested samples with fecal microbiota from both healthy and diseased individuals led to an even greater increase in the TPC compared to the non-fermented samples (Table 2). Notably, the TPC reached 6139 ± 458 g GAE/g in the samples fermented with microbiota from healthy adults (*p* < 0.0001) and 8375 ± 1388 g GAE/g with microbiota from healthy children (*p* < 0.001), whereas the non-fermented samples remained at 1657 ± 34 g GAE/g. These findings suggest that fermentation with gut microbiota, particularly from healthy individuals, significantly enhances the release of phenolic compounds, underscoring the substantial role of microbial metabolism in polyphenol biotransformation.

To assess the impact of fermentation with gut microbiota from healthy and diseased individuals on the TPC of the pomegranate extract, a comparative analysis was conducted across different treatment groups. No significant differences were observed in the TPC of the pomegranate extract fermented with microbiota from diseased individuals, except for the samples fermented with microbiota from celiac adults, which showed a statistically significant increase (*p* < 0.0001).

### 2.2. Radical Scavenging Activity of the Pomegranate Extract Before and After Digestion and Fermentation Processes

The radical scavenging capacity of the pomegranate extract before and after digestion and fermentation was evaluated using DPPH and ABTS+ assays. In its non-digested form, the extract exhibited antioxidant activity equivalent to 1996 ± 54 mg Trolox/g (TEAC_DPPH_) and 4994 ± 127 mg Trolox/g (TEAC_ABTS_). As shown in Table 1, in vitro digestion led to a significant increase in TEAC_DPPH_ (2504 ± 23 mg Trolox/g, *p* = 0.0013) and a notable decrease in TEAC_ABTS_ (2198 ± 76 mg Trolox/g, *p* = 0.0001).

Following in vitro fermentation, both TEAC_DPPH_ and TEAC_ABTS_ values significantly declined compared to the non-fermented samples (*p* < 0.0001, Table 2). Specifically, TEAC_DPPH_ dropped from 2504 ± 23 mg Trolox/g to 175 ± 11 mg Trolox/g after fermentation with microbiota from healthy adults and to 198 ± 31 mg Trolox/g with microbiota from healthy children. Similarly, TEAC_ABTS_ decreased from 2198 ± 76 mg Trolox/g to 162 ± 29 mg Trolox/g and 199 ± 21 mg Trolox/g, respectively. Notably, microbiota from diseased individuals (obese and celiac subjects) did not significantly alter TEAC_DPPH_ or TEAC_ABTS_ values compared to those observed after fermentation with microbiota from healthy adults and children.

### 2.3. Reducing Power of the Pomegranate Extract Before and After Digestion and Fermentation Processes

The FRAP assay indicated that the non-digested pomegranate extract exhibited a TEAC_FRAP_ value of 1924 ± 30.8 mg Trolox/g. As shown in Table 1, simulated in vitro digestion led to a slight but significant increase in TEAC_FRAP_ (2491 ± 175 mg Trolox/g, *p* = 0.0373). However, in vitro fermentation resulted in a significant reduction in TEAC_FRAP_ compared to the non-fermented samples (*p* < 0.0001, Table 2). Specifically, TEAC_FRAP_ decreased to 251 ± 15.3 mg Trolox/g in the samples fermented with microbiota from healthy adults and to 281 ± 27.7 mg Trolox/g in those fermented with microbiota from healthy children.

Among the diseased subjects, fermentation with microbiota from obese (201 ± 17 mg Trolox/g, *p* < 0.05) and celiac adults (188 ± 21 mg Trolox/g, *p* < 0.01) resulted in significantly lower TEAC_FRAP_ values compared to the samples fermented with microbiota from healthy adults (251 ± 15 mg Trolox/g). In contrast, no significant differences were observed in TEAC_FRAP_ between the samples fermented with microbiota from obese children (284 ± 31.3 mg Trolox/g) or celiac children (267 ± 10 mg Trolox/g) compared to their healthy counterparts (281 ± 27.7 mg Trolox/g).

### 2.4. SCFA Quantification After In Vitro Digestion and Fermentation

**Figure 1:**
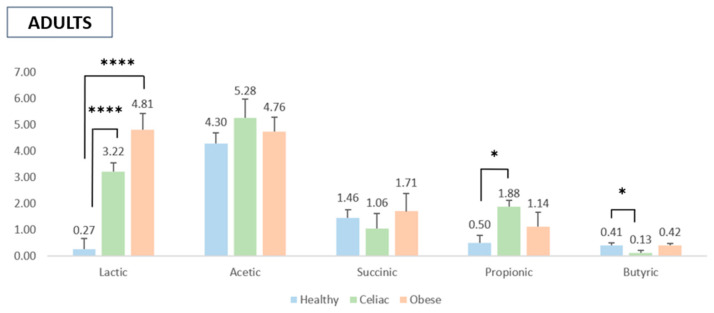
Production of SCFAs by the gut microbiota of adults upon addition of the digested pomegranate extract. The values are expressed in mmol/L as the net increase in the SCFA levels, determined by subtracting the SCFA values of the blank controls (fecal samples fermented without the pomegranate extract) from those of the pomegranate-containing samples. Data are presented as the means ± SD. Note: * *p* < 0.05 and **** *p* < 0.0001.

The analysis of SCFA production by gut microbiota isolated from healthy, obese, and celiac subjects in response to the digested pomegranate extract revealed significant differences between adults and children, likely reflecting variations in gut microbiota composition between these age groups. Notably, the gut microbiota of celiac and obese adults produced significantly higher levels of lactic acid following the addition of the digested pomegranate extract compared to healthy adults, whose lactate levels remained relatively unchanged (Figure 1). This suggests that pomegranate extract does not substantially affect lactate production in the gut microbiota of healthy adults compared to the control sample (i.e., fecal material without the pomegranate extract).

Although no statistically significant differences were observed among the three adult groups (healthy, obese, and celiac), a slight increase in acetic acid production and, to a lesser extent, succinic acid was noted after pomegranate extract supplementation. Additionally, the extract enhanced propionic acid production in celiac and obese subjects, with a statistically significant increase observed only between celiac and healthy adults. Lastly, a minor elevation in butyric acid levels was recorded in the microbiota of healthy and obese adults compared to the control samples (Figure 1).

**Figure 2:**
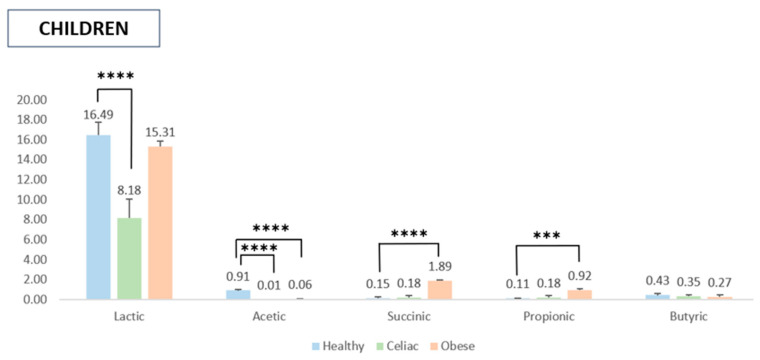
Production of SCFAs by the gut microbiota of children upon addition of the digested pomegranate extract. Values are expressed in mmol/L as the net increase in the SCFA levels, determined by subtracting the SCFA values of the blank controls (fecal samples fermented without the pomegranate extract) from those of the pomegranate-containing samples. Data are presented as the means ± SD. Note: *** *p* < 0.001 and **** *p* < 0.0001.

Figure 2 presents the SCFA production patterns in the pediatric population. The addition of the digested pomegranate extract during fermentation resulted in a significant increase in lactic acid production, whereas the levels of other SCFAs remained largely unchanged. Notably, acetic acid concentrations were higher in the samples treated with gut microbiota from healthy children compared to those from the children with pathological conditions. Regarding succinic and propionic acids, the gut microbiota of obese children exhibited significantly higher production levels compared to the reference group. However, statistical analysis did not reveal significant differences in butyric acid production among the three experimental groups.

### 2.5. Quali–Quantitative Analysis of Urolithin A After In Vitro Fermentation

**Figure 3:**
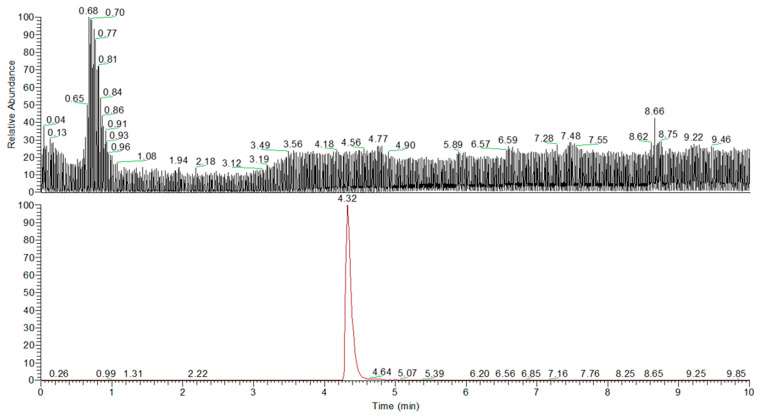
Total ion chromatogram (TIC) of the Uro-A standard obtained using UHPLC-Q-Orbitrap HRMS and extracted ion chromatogram (EIC) of Uro-A, identified at 4.32 min, corresponding to its exact mass (*m*/*z* = 227.03465).

**Figure 4:**
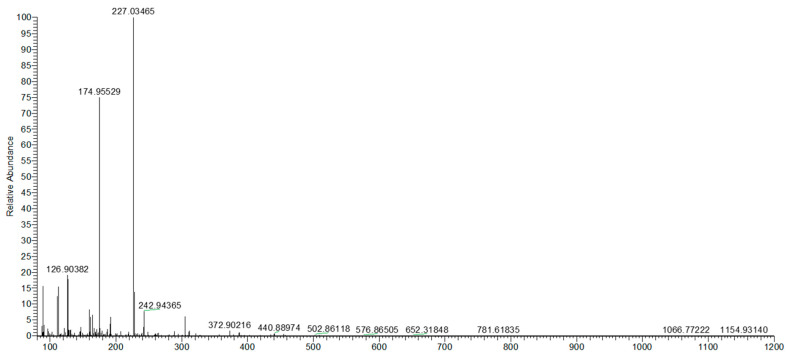
Full mass spectrum acquired in the scan range of *m*/*z* 80–500 in the negative ion mode (ESI–) of the Uro-A standard corresponding to *m*/*z* 227.03465.

The quantification of Uro-A was performed using a calibration curve prepared with a pure standard (purity ≥ 90%) dissolved in methanol containing 0.1% formic acid, with a concentration range of 0.01 to 5.0 mg/L. Data are presented in Figure 3 and Figure 4.

The UHPLC Q-Orbitrap HRMS analysis was conducted on a total of 36 samples to identify and quantify the presence of urolithins in the fermentation liquids produced by the gut microbiota of healthy, obese, and celiac subjects. The results revealed that only Uro-A was detected in one group of samples (digested pomegranate extract treated with gut microbiota isolated from healthy adults) at a concentration of 4.2 ng/mg.

The detection of Uro-A only in the samples from healthy subjects implies that in these experimental conditions, the ability to produce this metabolite may be limited or absent in obese and celiac subjects and in children.

## 3. Discussion

In this study, a pomegranate extract was subjected to simulated in vitro digestion using predefined protocols, followed by an assessment of its antioxidant profile (i.e., TPC, radical scavenging activity, and reducing power). The digested sample was then fermented with gut microbiota isolated from the fecal material of six donor groups (healthy adults and children, obese adults and children, and celiac adults and children). The digested and fermented samples were analyzed for their antioxidant profile and their capacity to modulate gut microbiota functionality in terms of SCFA production. Additionally, Uro-A quantification was performed after the in vitro fermentation process. The results of these experiments were compared with those obtained from the control samples prepared using the same digestion and fermentation protocols without the addition of the pomegranate extract.

As far as the TPC is concerned, our results are aligned with those obtained by Derakhshan et al. [23], who studied the TPC in ethanolic extracts from different parts of pomegranate, and Ardekani et al. [24], who analyzed the TPC in nine different pomegranate cultivars. The in vitro digestion of the pomegranate extract led to a significant increase in the TPC. These results are in accordance with those obtained by Fawole et al. [25] who observed a significant increase in the TPC following in vitro digestion of pomegranate juice, especially during the gastric phase. These results agree with previous studies on other fruits, showing the same pattern of increase in the TPC following in vitro digestion [26,27,28]. It can be assumed that the increase in the TPC after in vitro digestion could be attributed to the acidic hydrolysis of phenolic glycosides to their aglycones [29], as previous research on pomegranate extracts showed that most of the polyphenols in the extracts are in glycosidic forms [4]. In addition, pomegranate extract is rich in hydrolysable tannins, which are known to bind tightly with dietary fibers [30]. Digestion processes help to release phenolic compounds from the food matrix (such as fibers) and make them accessible for absorption from the gastrointestinal tract or to be metabolized by gut microbiota [31,32]. Contrarily, Perez-Vicente et al. [33] did not observe any significant effect of the gastric and duodenal digestion process on the TPC in the pomegranate extract. This result might be related to the difference in food matrix characteristics (i.e., pomegranate juice) and experimental conditions for in vitro digestion. Mosele et al. [34] observed an increase in the TPC for the pomegranate extract and a slight decrease in the TPC for the pomegranate juice, suggesting the impact of the food matrix on polyphenol bioaccessibility.

Regarding the antioxidant profile, simulated digestion showed a slight increase in the TEAC_DPPH_ and TEAC_FRAP_ values, while the TEAC_ABTS_ values were significantly decreased. In the present study, it was found that the antioxidant capacity of pomegranate was not correlated with the TPC of the extract. In the literature, it is well-established that a plant extract’s antioxidant activity is not only dependent on the polyphenol content, but also on their structure (i.e., number of hydroxyl groups, number and nature of substitutions on rings B and C, and degree of polymerization) [35,36].

With regard to the effect of fermentation on the bioaccessibility of pomegranate polyphenols, to the best of our knowledge, no data are available on the fermentation of pomegranate extract by human gut microbiota obtained from fecal material of healthy and diseased subjects. The present study showed a significant increase in the TPC of the digested pomegranate extract after fermentation with the fecal samples of healthy and diseased subjects. These results were expected, as during the fermentation process, key fermentable microbes can release enzymes such as tannases, glycosidases, esterases, and phenolic acid decarboxylases, which support the release of bound phenolic compounds from the food matrix, thus increasing the concentration of free polyphenol compounds, which are detectable with the Folin–Ciocalteu assay [37]. In such fruits as pomegranate, gut microbes could degrade ellagitannins to some intermediates, such as punicalin and gallagic acid, and eventually into EA [38]. Some fermentative strains (such as *Streptococcus thermophilus*) are known to transform EA to a new metabolite Uro-A, that may increase the TPC [39]. Unlike the TPC, the results of the antioxidant capacity of the pomegranate extract after fermentation with the fecal samples from healthy and diseased subjects were not as expected. A significant decrease in TEAC_DPPH_, TEAC_ABTS_, and TEAC_FRAP_ was observed despite a significant increase in the TPC. Singh et al. [40] reported that the interaction between epithelial cells and commensal bacteria increases the rapid production of ROS to maintain gut barrier protection. Therefore, fecal materials obtained from healthy and diseased adults and children may contain high levels of ROS, which could be scavenged by the anti-radical compounds present in digested pomegranate extract. After interacting with the fecal material, the extract showed a lower residual anti-radical activity than that observed post-digestion alone, likely due to the reduction of ROS levels.

As far as SCFAs are concerned, this study demonstrated that fermentation of the in vitro digested pomegranate extract modulated SCFA production by gut microbiota isolated from diseased subjects, particularly lactic acid in both adults and children. Fermentation with microbiota isolated from adult subjects significantly increased the concentration of lactic acid in diseased individuals (i.e., celiac and especially in obese adults) as compared to healthy ones. In addition, slight effects were observed on acetic and succinic acids when microbiota from healthy, obese, and celiac subjects was treated with the digested pomegranate extract. Notably, fermentation showed statistically significant effects on propionic acid in celiac subjects compared to healthy adults, while a slight increase in butyric acid concentration was noted in healthy and obese subjects. In the microbiota of the obese children, fermentation of the pomegranate extract resulted in a significant increase in lactic, succinic, and propionic acid production. The acetic concentration was increased only in the healthy subjects, while no significant effects on butyric acid were observed in both groups. It is well-known that the gut microbiota plays a crucial role in the fermentation of some dietary compounds, which are inert to digestive enzymes in the upper gastrointestinal tract and are converted into SCFAs (acetate, propionate, and butyrate). In fact, some of these SCFAs are used by enterocytes as a source of energy and exert beneficial effects (i.e., regulatory effects on immunity, metabolism, and gastrointestinal physiology) [41]. Specifically, butyrate promotes intestinal cell regeneration and protection, mucus production, a decrease in hypercholesterolemia levels, and the release of hormones and/or neurotransmitters that control intestinal motility and insulin resistance [42]. In addition to dietary fibers, polyphenols have been recognized as a new class of prebiotics that meet the criteria to be classified as a prebiotic substrate, such as resistance to host digestion, the capacity to be fermented by the gut microbiota, and the ability to modulate gut microbiota functionality [43]. The effect of pomegranate extract on the modulation of the synthesis of SCFAs can be attributed to its high content of polyphenols.

Moreover, the interaction of the gut microbiota with the immune system of the host is one of the key elements explaining the pathogenesis of a large spectrum of diseases, including obesity and celiac disease [44]. Obesity is generally associated with reduced microbial diversity, SCFA-synthesizing bacteria, and SCFA concentration, thus influencing adipose tissue composition, fat mass gain, chronic low-grade inflammation, and insulin resistance [45]. Some in vivo studies suggest that supplementation with SCFAs may reduce body weight, improve insulin sensitivity, and reduce chronic low-grade inflammation [46,47,48,49,50]. Similarly, patients with celiac disease exhibit specific gut microbiota profiles, characterized by reduced microbial diversity and alterations in SCFA concentrations, which may reflect impaired fermentation processes and contribute to intestinal inflammation [51,52,53]. A decrease in *Bacteroides, Bifidobacterium* species, and *Parabacteroides* in celiac disease, with an increased abundance of *Escherichia, Helicobacter*, and *Candida albicans*, has been reported in the literature [54]. Considering this evidence, gut microbiota modulation can be considered one of the most important targets in the prevention and treatment of chronic pathologies. The current study, with notable effects on lactic acid production, indicated that pomegranate extract may have pronounced effects on the activity of lactic acid-producing bacteria [55]. A study by Mosele et al. [56] showed no significant effects of pomegranate juice on the gut microbiome composition in healthy adults, though the authors observed effects on health-promoting phenolic metabolites, such as catechol and 3-phenylpropionic acid. Another study demonstrated that the fermentation of fecal samples from healthy subjects with pomegranate byproducts resulted in an enhanced growth of *Lactobacillus* and *Bifidobacterium* species, as well as an increased production of SCFAs, such as acetate, propionate, and butyrate, indicating potential prebiotic benefits of these byproducts [14]. In an animal study, Song et al. demonstrated attenuation of obesity, improvement of insulin resistance, and modulation of gut microbiota in mice supplemented with pomegranate peel extract [57]. The results showed a decrease in body weight and the insulin resistance index by 27.46% and 46.07%, respectively. The gut microbiota analysis indicated enrichment of *Bacteroides* with a decreased abundance of *Firmicutes* and *Proteobacteria* in pomegranate-supplemented mice. In addition, pomegranate also increased the abundance of *Akkermansia*, *Anaerotruncus*, *Lachnoclostridium*, and *Parabacteroides* species. In another study [58], pomegranate extract and Uro-A mitigated inflammatory responses and preserved colonic structure by modulating gut microbiota and downregulating the key inflammatory pathways in a rat model of colitis. Both pomegranate extract and Uro-A decreased inflammatory markers in colon mucosa, including inducible nitric oxide synthase (iNOS), prostaglandin E synthase, prostaglandin E, and cyclooxygenase-2. The fecal microbiota analysis indicated an increase in *Lactobacillus* and *Bifidobacterium* species in rats treated with pomegranate extract (250 mg/kg/day) and Uro-A (15 mg/kg/day) for 10 days, as compared to the control.

In recent years, there has been a notable increase in in vivo studies to investigate the biological effects of urolithins and confirm the health benefits associated with the consumption of ellagitannin (ET)-rich foods [59]. This growing interest is partly driven by the well-established fact that the bioavailability of ETs and EA is very low, primarily due to their poor absorption in the gastrointestinal tract. As a result, these compounds undergo extensive microbial metabolism in the colon, leading to the production of urolithins, which are thought to be the key bioactive metabolites responsible for the observed health benefits.

In our experimental conditions, Uro-A was detected at low concentrations exclusively in the fecal microbiota samples isolated from healthy adults following the in vitro simulated digestion and fermentation of the pomegranate extract. The most plausible explanation is that the fecal material of children and diseased subjects lacks sufficient microorganisms capable of converting EA into Uro-A. Moreover, according to Mena et al. [60], the digestion process can significantly affect the stability and availability of EA, potentially resulting in a considerable loss and reducing its availability for fermentation by gut microbiota. In fact, they demonstrated that gastric digestion significantly reduces the available amount of EA due to the highly acidic pH of the stomach, with a decrease from 45% to 5% during this phase. Although a slight recovery was observed in the intestinal phase, the EA available for fermentation was still reduced compared to the beginning of digestion, and extensive degradation also occurred for Uro-A and EA during the colonic step [60]. In our experiments, the simulated digestive phase may have degraded a significant amount of ETs, leaving an insufficient quantity for effective conversion into urolithins during fecal fermentation. This idea is further supported by the fact that many in vitro studies, such as those conducted by He et al. [61], have focused on assessing the capacity of the gut microbiota isolated from fecal material to metabolize ellagic acid into urolithins. In contrast, our findings differ from those of human studies, which have demonstrated that the intake of ellagitannin-rich foods, where digestion occurs naturally, enables the identification and quantification of the resulting metabolites in biological samples such as urine, plasma, and feces [62,63,64]. For instance, Mosele et al. showed that urolithins are produced from pomegranate extracts following in vitro digestion and fermentation, with considerable variability in the amount of urolithin derivatives due to differences in individual colonic microbiota [34]. Similarly, Tan et al. investigated the bioavailability of rambutan peel polyphenols (RPPs) and found that ellagic acid could be transformed into urolithins during in vitro gastrointestinal digestion, Caco-2 cell transport, and colonic fermentation, influenced by the action of intestinal microbiota [65].

In the present study, the simulated in vitro fermentation process ended after 24 h of incubation with human fecal material, but this may not have provided sufficient time for the complete conversion of EA into Uro-A, as reported by Garcia-Villalba et al., who observed that Uro-A is detected after 14 h and increases over time, stabilizing around 42 h during in vitro fecal fermentation of EA [63]. Similarly, Dacrema et al. demonstrated in vivo that while Uro-A levels in CD1 mouse plasma were relatively low after 24 h, they increased over time, reaching higher concentrations up to two weeks post-intake, indicating a delayed conversion process [66].

It is also known that the efficiency of EA conversion into urolithins can vary significantly between individuals, with differences in urolithin production profiles between “producers” and “non-producers,” depending on the presence or absence of specific bacterial groups [67,68]. It is possible that the composition of the donors’ fecal microbiota influenced the ability to convert EA into Uro-A, contributing to the absence of detectable metabolites. This may reflect the presence of microbial species that are not directly involved in the production of certain urolithins.

This study has its own limitations and strengths. The main limitation is the sole use of an in vitro experimental model, as unlike in vivo and clinical trials, in vitro experimental models are not true representatives of human systems. The simulated in vitro digestion and fermentation processes do not perfectly mimic what occurs in the human intestine. The in vitro conditions may not accurately reflect the complex and dynamic environment of the gut. First of all, the metabolic activity of gut microbes and urolithin production may vary depending on the availability of additional carbon sources, suggesting that future studies evaluate the impact of co-fermentation with other nutrients, such as prebiotics, for example, inulin, to better simulate real dietary conditions. Factors such as pH levels, enzyme activity, interactions with host tissues, and the presence of different microbial populations can significantly influence metabolic processes. Moreover, the time required for complete Uro-A production may vary across individuals and experiments, suggesting that longer incubation periods or alternative methodologies might be necessary to better assess urolithin production in in vitro experiments. Given the individual variability in the gut microbiota composition, future studies should also focus on characterizing the specific microbial taxa responsible for urolithin production and the conditions that optimize this metabolic conversion. In addition, the SCFA concentration in different fecal samples from the healthy and diseased subjects was determined, but the effect of the pomegranate extract on the diversity and activity of individual gut microbiota strains was not assessed. However, the impact of pomegranate extract on the functionality of the gut microbiota from healthy and diseased subjects (involving both adults and children) was determined for the first time, which is the major strength of the current investigation.

## 4. Materials and Methods

### 4.1. Chemicals and Reagents

Iron (III) chloride hexahydrate, 2,4,6-tri(2-pyridyl)-s-triazine (TPTZ), sodium acetate, 2,2-diphenyl-1-picrylhydrazyl hydrate (DPPH), 2,2′-azinobis(3-ethylbenzothiazoline)-6-sulfonic acid (ABTS), hydrochloric acid, methanol, 6-hydroxy-2,5,7,8-tetramethylchromane-2-carboxylic acid (Trolox), Folin–Ciocalteu reagent, sodium carbonate, gallic acid, potassium dihydrogen phosphate, potassium chloride, magnesium chloride hexahydrate, sodium chloride, calcium chloride dihydrate, sodium bicarbonate, ammonium carbonate, hydrochloric acid, salivary alpha-amylase, pepsin from porcine gastric mucosa, bile extract from porcine, sodium dihydrogen phosphate, sodium sulfide, tryptone, cysteine, resazurin, acetic acid, propionic acid, and butyric acid were obtained from Sigma-Aldrich (Germany). Analytical-grade methanol (MeOH), water, and formic acid (FA) were obtained from Carlo Erba Reagents (Milan, Italy). Urolithin A (C_13_H_8_O_4_), with a purity of ≥90%, was acquired from Merck Group (Darmstadt, Germany) and used as a reference standard for quantitative analysis. Urolithin A was dissolved in methanol to produce a stock solution at a concentration of 1 mg/mL. By starting from an individual stock solution, the calibration curve was generated by diluting it in methanol containing 0.1% formic acid, resulting in a final concentration ranging from 0.01 to 5.0 mg/L.

The pomegranate fruit was macerated in water and chopped into small pieces. Subsequently, the extract was obtained by mixing a hydroalcoholic solution, specifically in a 70:30 ethanol/water ratio, in a single-phase extraction using ultrafiltration absorbent membranes (0.01–0.1 μm) to obtain a particle-free liquid extract. Finally, as reported by Ullah et al. [4], this extract was then subjected to a spray-drying process to obtain a dry extract.

### 4.2. In Vitro Digestion

The pomegranate extract underwent in vitro gastrointestinal digestion to simulate physiological human intestinal processes, following an adaptation of the protocol by Minekus et al. [69]. The digestion process consisted of three phases: oral, gastric, and intestinal. Initially, 5 g of the sample was mixed with 5 mL of simulated salivary fluid containing α-amylase (150 U/mL) and incubated at 37 °C for 5 min under continuous oscillation. Subsequently, 10 mL of simulated gastric fluid containing pepsin (4000 U/mL) were added, and the pH was adjusted to 3. The mixture was maintained at 37 °C for 2 h under oscillation. Finally, 20 mL of simulated intestinal fluid containing pancreatin (200 U/mL) and bile salts (20 mM) were introduced, and the pH was raised to 7. The sample was then incubated at 37 °C for another 2 h. To halt the enzymatic activity, the mixture was placed on ice for 15 min. The supernatant, representing the bioavailable fraction for small intestine absorption, was collected and stored at −20 °C for subsequent antioxidant capacity analysis. Meanwhile, the pellet, representing the undigested fraction destined for colonic fermentation, was directly used for in vitro fermentation. A control sample containing only enzymes and salts was also processed. All experiments were conducted in triplicate, generating three independent digestion liquids for subsequent fermentation.

### 4.3. In Vitro Fermentation

This process was conducted following the protocol described by Pérez-Burillo et al. [70], using fecal material from various Caucasian donors. Adult donors (aged 21–42 years) included healthy individuals (BMI, 18.5–24.99), obese individuals (BMI ≥ 30), and celiac individuals (BMI within the normal range, 18.5–24.99). Pediatric donors (aged 6–12 years) included healthy children (BMI, 5th–85th percentile), obese children (BMI > 95th percentile), and celiac children (BMI, 5th–85th percentile). Fecal material was collected using a sterile container (Fecotainer, AT Medical) and gloves, with the help of a disposable sterile spoon. Once collected, fecal material was stored by the volunteer at home in a refrigerator and transported to the laboratory in a cooler bag within 24 h. Upon arrival at the laboratory, fecal samples from three donors for each experimental condition were pooled to account for interindividual variability and used for in vitro fermentations. The study adhered to the guidelines of the Declaration of Helsinki and was approved by the Ethics Committee of the University of Granada (protocol code 1080/CEIH/2020) for the adult participants and by the Scientific Committee of the University Hospital “Attikon” (decision No. 546/1-10-2020) for the pediatric participants. None of the donors had taken antibiotics within the previous three months.

In vitro fermentation was performed in 15 mL centrifuge tubes. Each tube contained 500 mg of the solid digestion residue along with 10% of the final digestion volume. A control tube was also included, which did not contain the pomegranate extract. Additionally, each tube was supplemented with 7.5 mL of the fermentation growth medium (comprising 14 g/L of peptone, 312 mg/L of cysteine, 312 mg/L of hydrogen sulfide, and 0.1% *v*/*v* resazurin) and 2 mL of the fecal inoculum (prepared by suspending the fecal material in phosphate-buffered saline, 33% *w*/*v*).

To maintain anaerobic conditions, nitrogen gas was bubbled into the tubes, which were then incubated at 37 °C under oscillation for 20 h. Microbial activity was halted by placing the tubes on ice for 15 min. The samples were then centrifuged at 6000× *g* for 10 min, and the resulting supernatant was aliquoted and stored at −20 °C for subsequent antioxidant capacity and SCFA analysis. This process generated three fermentation liquids per experimental condition.

### 4.4. Antioxidant Assays

Both the supernatant from in vitro digestion and the supernatant from in vitro fermentation were analyzed for their antioxidant capacity. The total antioxidant capacity was determined as the sum of the antioxidant capacities of these two fractions. To ensure accuracy, respective blanks (containing only chemicals, enzymes, and the inoculum) were used to correct the antioxidant capacity values for each method. To evaluate the impact of digestion and fermentation on the TPC and antioxidant capacity of the pomegranate extract, the digested samples were compared with the non-digested samples, while the fermented samples (from healthy subjects) were compared with the non-fermented (digested) samples.

All experiments were performed in triplicate for both digestion and fermentation liquids.

#### 4.4.1. Total Phenolic Content (TPC)

The TPC of the pomegranate samples was determined using the Folin–Ciocalteu assay, following the protocol of Moreno-Montoro et al. [71]. An aliquot of 30 μL of the digestion or fermentation supernatant was added in triplicate to a 96-well plate (Cytation, Agilent Technologies Inc., Santa Clara, CA, USA) and mixed with 60 μL of 10% (*w*/*v*) sodium carbonate, 195 μL of ultra-pure water, and 15 μL of the Folin–Ciocalteu reagent. The antioxidant reaction was monitored for 60 min at 37 °C. A calibration curve was prepared using gallic acid at concentrations ranging from 0.01 to 1.00 mg/mL. The results were expressed as mg gallic acid equivalent per gram (mg GAE/g) of pomegranate extract.

#### 4.4.2. DPPH Assay

The DPPH assay was performed following the protocol of Yen and Chen [72]. Twenty μL of the digestion or fermentation supernatant were mixed with 280 μL of a freshly prepared DPPH reagent in a 96-well plate. The DPPH reagent was prepared daily by dissolving 74 mg of DPPH in 1 L of methanol. The reaction was monitored for one hour at 37 °C using a Cytation 5 plate reader (Agilent Technologies Inc., Santa Clara, CA, USA). Each sample was analyzed in triplicate. A calibration curve was prepared using Trolox (0.01–0.4 mg/mL), and the results were expressed as mg Trolox equivalent per gram of pomegranate extract.

#### 4.4.3. ABTS Assay

The ABTS+ assay was performed following the method described by Re et al. [73], with slight modifications. The ABTS+ solution was prepared by reacting a 7 mM ABTS+ stock solution with 2.45 mM potassium persulfate and allowing the mixture to stand in the dark at room temperature for 12–16 h before use. Before the assay, the ABTS+ solution was diluted with 5 mM phosphate-buffered saline (pH 7.4) to achieve an absorbance of 0.70 ± 0.02 at 730 nm. Then, 10 μL of the sample were added to 4 mL of the diluted ABTS+ solution, and absorbance was measured after 20 min using a Cytation 5 plate reader (Agilent Technologies Inc., Santa Clara, CA, USA). A calibration curve was prepared using the Trolox stock solution, and the results were expressed as mg Trolox equivalent per gram of sample.

#### 4.4.4. FRAP Assay

The FRAP assay was performed following the method of Benzie and Strain [74] using a Cytation 5 microplate reader (Agilent Technologies Inc., Santa Clara, CA, USA). For each sample, including the digestion and fermentation supernatants, 20 μL were mixed with 280 μL of a freshly prepared FRAP reagent in a 96-well plate. The FRAP reagent consisted of 0.3 M sodium acetate buffer (pH 3.6), ferric chloride solution, and 40 mM 2,4,6-tri(2-pyridyl)-s-triazine (TPTZ) solution in a ratio of 10:1:1. The reaction was conducted at 37 °C, and absorbance was measured at 595 nm every 30 s for 30 min. Each sample was analyzed in triplicate to ensure accuracy. A calibration curve was prepared using Trolox at concentrations ranging from 0.01 to 0.4 mg/mL, and the results were expressed as mg Trolox equivalent per gram of pomegranate extract.

### 4.5. Analysis of Short-Chain Fatty Acids (SCFAs)

The preparation of samples for chromatographic analysis involved centrifuging the fermentation supernatant at 13,300 rpm for 5 min. The supernatant was then filtered through a 0.22 μm filter and diluted in a 1:10 ratio with 1 M hydrochloric acid, following the protocol described by Panzella et al. [75]. The analysis was performed using an Agilent Poroshell 120 SB-Aq column (3 × 150 mm, 2.7 μm) with 5 mM sulfuric acid as the mobile phase under isocratic elution at a flow rate of 0.5 mL/min. A sample injection volume of 5 µL was used, and both the column and refractive index detector (RID) temperatures were maintained at 35 °C. Chromatographic analysis was conducted in triplicate for each sample using an Agilent 1290 Infinity II system (Agilent Technologies Inc., Santa Clara, CA, USA).

For quantification, calibration was performed using external standards of lactic, acetic, succinic, propionic, *n*-butyric, and isobutyric acids. The *n*-butyric and isobutyric acids were quantified together. The total SCFAs were calculated as the sum of acetic, propionic, and butyric acids, and the results were expressed in mmol of each acid per liter of the fermentation supernatant. The SCFA analysis was carried out in triplicate for each experimental condition, including healthy, obese, and celiac subjects from both adult and pediatric groups.

### 4.6. Extraction of Urolithin A

The extraction of urolithin A was carried out following the protocol described by García-Villalba et al. [76], with some modifications. Five milligrams of each freeze-dried sample were placed in a 50 mL tube, and 5 mL of methanol acidified with 0.1% formic acid were added. The mixture was vortexed for 3 min using a MIX ARGOlab Vortex Mixer GB22008013. Following this, the sample was sonicated for 10 min in an ultrasonic bath (LBS 1-6 Lt; FALC, Treviglio (BG), Italy) and then centrifuged at 5000 rpm for 5 min at 4 °C using an SL16R centrifuge (Thermo Fisher Scientific, Dreieich, Germany). The supernatant was collected and filtered through a 0.2 μm nylon syringe filter. Finally, ultrahigh-performance liquid chromatography coupled with high-resolution mass spectrometry (UHPLC Q-Orbitrap HRMS; Thermo Fisher Scientific, Dreieich, Germany) was used for the analysis.

### 4.7. UHPLC Q-Orbitrap HRMS Analysis of Urolithin A

The qualitative and quantitative measurement of urolithin A was conducted using ultrahigh-performance liquid chromatography coupled with high-resolution mass spectrometry (UHPLC Q-Orbitrap HRMS). Mass spectrometry analysis was performed on a Q-Exactive mass spectrometer (Thermo Fisher Scientific, Waltham, MA, USA) equipped with an electrospray ionization (ESI) source. The analysis was carried out in the negative ionization mode, targeting a mass range of 80 to 500 *m*/*z*. Data acquisition was performed in the full-ion mass spectrometry (MS) mode, with the following key parameters: scan range of 80–500 *m*/*z*, resolution of 70,000 full width at half maximum (FWHM), microscan set to 1, automatic gain control (AGC) target of 1 × 10^6^, maximum injection time of 200 ms, sheath gas flow rate of 35, auxiliary gas flow rate of 10, spray voltage of 2.8 kV, capillary temperature of 375 °C, S-lens RF level of 50, and auxiliary gas heater temperature of 350 °C. A mass tolerance of 5 ppm was maintained for the identification and confirmation of urolithin A ions.

Chromatographic separation was performed using a UHPLC system (Dionex UltiMate 3000; Thermo Fisher Scientific, Waltham, MA, USA) equipped with a degassing unit, a quaternary pump, and an autosampler. A Kinetex F5 column (2.6 µm particle size, 100 mm × 2.1 mm) from Phenomenex (Torrance, CA, USA) was used, maintained at 20 °C. The mobile phase consisted of water (A) and methanol (B), both acidified with 0.1% formic acid. Optimal separation was achieved using the following gradient elution program: from 0 to 0.5 min at 100% A, followed by a decrease from 100% to 30% A between 0.5 and 1 min, and then further decreasing from 30% to 0% A from 1 to 7.8 min. The mobile phase was held at 0% A from 7.8 to 8.3 min, followed by re-equilibration back to 100% A from 8.3 to 10 min. The flow rate was set at 400 µL/min, with an injection volume of 5 µL. Data analysis was performed using Xcalibur software (version 3.1.66.19; Thermo Fisher Scientific).

### 4.8. Statistical Analysis

For the statistical analysis, the data were presented as the means ± standard deviation (SD). A paired t-test was utilized to determine the differences in the pomegranate extract before and after in vitro oral, gastric, and intestinal digestion processes. Additionally, statistical comparisons between the groups were conducted using one-way ANOVA followed by Dunnett’s post-hoc test for multiple comparisons to determine significance, which was set at *p* < 0.05. Concerning the antioxidant profile of the pomegranate extract, two different analyses were performed to assess the differences between the digested and fermented samples, and the differences between the healthy subjects (control group), obese, and celiac individuals after in vitro fermentation. The statistical analyses were performed using GraphPad Prism, version 8 (San Diego, CA, USA). Finally, the statistical analysis regarding the SCFAs was conducted using the SPSS statistical program. The mean values and standard deviations for each group were determined, and the statistical significance of the data was assessed using the non-parametric Kolmogorov–Smirnov analysis of variance test, followed by Bonferroni post-hoc tests to compare the samples that exhibited significant variation (*p* < 0.05).

## 5. Conclusions

This study highlights the antioxidant potential and gut microbiota-modulating effects of fermented pomegranate extract, demonstrating its capacity to enhance SCFA production, particularly lactic acid, in individuals with obesity and celiac disease. Given the critical role of gut microbiota in metabolic and gastrointestinal disorders, dietary interventions targeting microbial composition and activity represent a promising strategy for disease prevention and management. Notably, polyphenols and the gut microbiota share a bidirectional relationship: polyphenols influence microbial diversity and activity, while the gut microbiota transforms polyphenols into bioactive metabolites such as urolithins, enhancing their health benefits. Our findings support the potential prebiotic application of pomegranate extract in functional foods and dietary supplements, offering a natural approach to modulating the gut microbiota in at-risk populations.

However, this study is limited by the absence of next-generation sequencing (NGS) data, which would provide a deeper understanding of microbial shifts and specific bacterial taxa involved in polyphenol metabolism. Future research should integrate metagenomic and metabolomic approaches to further elucidate the mechanistic interactions between pomegranate polyphenols and the gut microbiota. Such insights could facilitate the development of personalized nutrition strategies and targeted functional foods for improved gut health and disease management.

## Figures and Tables

**Table 1:** Antioxidant capacity of the pomegranate extract before and after in vitro oro–gastro–intestinal digestion process.

Assay	Before In Vitro Digestion	After In Vitro Digestion	*p*-Value
TPC (g of GAE/kg of extract)	232 ± 16	1657 ± 34	<0.0001
TEAC_DPPH_ (g of Trolox/kg of extract)	1996 ± 54	2504 ± 23	0.0013
TEAC_ABTS_ (g of Trolox/kg of extract)	4994 ± 127	2198 ± 76	0.0001
TEAC_FRAP_ (g of Trolox/kg of extract)	1924 ± 31	2491 ± 175	0.0373

Values are expressed as the means ± standard deviation. The TPC is expressed in mg of GAE/g of extract, while TEAC_DPPH_, TEAC_FRAP_, and TEAC_ABTS_ are expressed in mg Trolox/g of extract.

**Table 2:** Antioxidant capacity of the pomegranate extract before and after in vitro digestion and fermentation with gut microbiota isolated from healthy, obese, and celiac subjects.

Assay	In Vitro Digested Samples	After In Vitro Fermentation					
		Adults			Children		
		Healthy	Obese	Celiac	Healthy	Obese	Celiac
TPC	1657 ± 34	6139 ± 458 ****	5889 ± 259 ^ns^	2914 ± 367 ****	8375 ± 1388 ***	8945 ± 1120 ^ns^	8195 ± 1180 ^ns^
TEAC_DPPH_	2504 ± 23	175 ± 11 ****	233 ± 30 ^ns^	241 ± 37 ^ns^	198 ± 31 ****	195 ± 31 ^ns^	203 ± 31 ^ns^
TEAC_ABTS_	2198 ± 76	162 ± 29 ****	107 ± 29 ^ns^	155 ± 23 ^ns^	199 ± 21 ****	193 ± 23 ^ns^	200 ± 31 ^ns^
TEAC_FRAP_	2491 ± 175	251 ± 15 ****	201 ± 18 *	188 ± 21 **	281 ± 28 ****	284 ± 31 ^ns^	267 ± 10 ^ns^

Values are expressed as the means ± standard deviation. The TPC is expressed in mg of GAE/g of extract, while TEAC_DPPH_, TEAC_FRAP_, and TEAC_ABTS_ are expressed in mg Trolox/g of extract. The samples fermented using fecal microbiota isolated from healthy adults and healthy children were compared with the non-fermented samples to determine the statistically significant change in the TPC values and antioxidant profile. Similarly, the samples fermented using fecal microbiota isolated from obese and celiac subjects were compared with those from healthy adults and children to evaluate the impact of the pomegranate extract on the TPC values and antioxidant capacity in diseased states. Note: ns, non-significant, * *p* < 0.05, ** *p* < 0.01, *** *p* < 0.001, **** *p* < 0.0001.

## Data Availability

The original contributions presented in the study are included in the article, further inquiries can be directed to the corresponding author.

## Abbreviations

The following abbreviations are used in this manuscript:

ABTS	2,2′-azinobis(3-ethylbenzothiazoline)-6-sulfonic acid
AP-1	Activator protein
COX	Cyclooxygenase
DPPH	2,2-diphenyl-1-picrylhydrazyl hydrate
EA	Ellagic acid
H_2_O_2_	Hydrogen peroxide
IL	Interleukins
NF-κB	Nuclear factor kappa B
PPARs	Peroxisome proliferator-activated receptors
SCFAs	Short-chain fatty acids
STAT	Signal transducer and activator of transcription
TNF-α	Tumor necrosis factor
TPC	Total phenolic content
TPTZ	2,4,6-tri(2-pyridyl)-s-triazine
Trolox	6-hydroxy-2,5,7,8-tetramethylchromane-2-carboxylic acid
UHPLC Q-Orbitrap HRMS	Ultrahigh-performance liquid chromatography coupled with high-resolution mass spectrometry
Uro-A	Urolithin A
